# Cervical spinal cord atrophy in amyotrophic lateral sclerosis across disease stages

**DOI:** 10.1002/acn3.51712

**Published:** 2023-01-04

**Authors:** Anna Nigri, Eleonora Dalla Bella, Stefania Ferraro, Jean Paul Medina Carrion, Greta Demichelis, Enrica Bersano, Monica Consonni, Antje Bischof, Mario Stanziano, Sara Palermo, Giuseppe Lauria, Maria Grazia Bruzzone, Nico Papinutto

**Affiliations:** ^1^ Neuroradiology Unit Fondazione IRCCS Istituto Neurologico Carlo Besta Milan Italy; ^2^ 3rd Neurology Unit and Motor Neuron Disease Centre Fondazione IRCCS Istituto Neurologico Carlo Besta Milan Italy; ^3^ School of Life Science and Technology, MOE Key Laboratory for Neuroinformation University of Electronic Science and Technology of China Chengdu China; ^4^ Department of Medical Biotechnology and Translational Medicine University of Milan Milan Italy; ^5^ Weill Institute for Neurosciences, Department of Neurology University of California San Francisco California USA; ^6^ Department of Neurology with Institute for Translational Neurology University Hospital Münster Münster Germany; ^7^ ALS Centre, “Rita Levi Montalcini” Department of Neuroscience University of Turin Turin Italy

## Abstract

**Objective:**

Spinal cord degeneration is a hallmark of amyotrophic lateral sclerosis. The assessment of gray matter and white matter cervical spinal cord atrophy across clinical stages defined using the King's staging system could advance the understanding of amyotrophic lateral sclerosis progression.

**Methods:**

We assessed the in vivo spatial pattern of gray and white matter atrophy along cervical spinal cord (C2 to C6 segments) using 2D phase‐sensitive inversion recovery imaging in a cohort of 44 amyotrophic lateral sclerosis patients, evaluating its change across the King's stages and the correlation with disability scored by the amyotrophic lateral sclerosis functional rating scale revised (ALSFRS‐R) and disease duration. A mathematical model inferring the potential onset of cervical gray matter atrophy was developed.

**Results:**

In amyotrophic lateral sclerosis patients at King's stage 1, significant cervical spinal cord alterations were mainly identified in gray matter, whereas they involved both gray and white matter in patients at King's stage ≥ 2. Gray and white matter areas correlated with clinical disability at all cervical segments. C3–C4 level was the segment showing early gray matter atrophy starting about 7 to 20 months before symptom onset according to our model.

**Interpretation:**

Our findings suggest that cervical spinal cord atrophy spreads from gray to white matter across King's stages in amyotrophic lateral sclerosis, making spinal cord magnetic resonance imaging an in vivo assessment tool to measure the progression of the disease.

## Introduction

Amyotrophic lateral sclerosis (ALS) is a fatal neurodegenerative disease^1^ caused by the progressive degeneration of both upper and lower motor neurons,^2^ resulting in progressive weakness of voluntary muscles up to death from respiratory failure within 2–5 years from clinical onset.^3^

Spinal cord (SC) degeneration is one of the hallmarks of ALS.^4^ Total cross‐sectional area (TCA) or volume of the cervical SC is significantly reduced in ALS patients compared to healthy subjects^5^, ^6^, ^7^, ^8^, ^9^, ^10^, ^11^, ^12^, ^13^ with atrophy more prominent in the caudal portion.^8^, ^10^, ^11^ The degree of cervical SC atrophy is predictive of shorter life expectancy^9^, ^14^ and worsening of respiratory functions at 12 months,^9^ with longitudinal studies showing significant SC tissue loss at 3 months' follow‐up,^9^, ^10^, ^15^, ^16^ mainly localized in the caudal segments of the cervical SC.^10^, ^11^

Although more refined in vivo SC studies are needed to better understand the pathophysiology of ALS, the majority of magnetic resonance imaging studies have been limited to the investigation of the SC in its entirety, without differentiating the gray and white matter, because of the challenges posed by SC imaging (e.g., small dimension, strong magnetic susceptibility effects, motion in the area due to swallowing and respiration).^4^ The few studies conducted in small cohorts of patients and evaluating the differential impact of ALS on cervical SC gray and white matter showed severe atrophy of both regions in the caudal portion.^8^, ^17^, ^18^

Recent advances in SC imaging offer new perspectives for the quantitative assessment of neurodegeneration in ALS, enabling the separate measure of cross‐sectional gray matter (GMA) and white matter (WMA) areas. Together with the T2*‐weighted protocols recently used in the field, Phase‐sensitive inversion recovery (PSIR) is a well‐established technique to reliably quantify the SC TCA, GMA, and WMA in the clinical settings thanks to fast acquisition times, high contrast‐to‐noise ratio between SC tissues and cerebrospinal fluid, and relatively low sensitivity to magnetic and motion artifacts.^19^, ^20^ As evidence of its ability to selectively quantify GMA and WMA, PSIR has been successfully applied in the study of gray and white matter atrophy in large cohorts of patients with multiple sclerosis.^21^ Moreover, PSIR was already employed to investigate SC GMA and WMA atrophy in motor neuron disease in a small cohort of subjects as a proof of concept of the clinical applicability of this magnetic resonance imaging sequence.^18^

In this study, we sought to identify a possible SC imaging marker of disease progression that can be easily employed in a clinical context. To this aim, we assessed the pattern of gray and white matter atrophy along the cervical portion of the SC (from C2 to C6 vertebral segments) in a large cohort of early diagnosed sporadic ALS patients employing a 2D PSIR protocol. We investigated the gray and white matter changes across patients' clinical stages classified by the King's staging system^22^ and their correlation with clinical measures. Finally, a linear regression model inferring the potential onset of cervical gray matter atrophy was developed.

## Material and Methods

### Participants

Between 2019 and 2020, 48 sporadic patients diagnosed with probable or definite ALS based on El Escorial revised criteria,^23^ and 17 healthy participants were recruited at the Motor Neuron Diseases Centre of the Fondazione IRCCS Neurological Institute “Carlo Besta” in Milan. Exclusion criteria were clinical and/or neuroimaging evidence of cerebrovascular diseases and neurological or psychiatric diseases.

All patients underwent genetic assay for *C9orf72, SOD1, FUS, OPTN, TARDBP* mutations. The presence of a genetic mutation was considered an additional exclusion criterion for this study. An in‐depth clinical assessment was performed in all patients for the evaluation of the type of onset (bulbar vs. spinal) and the involvement of clinical damage of upper and lower motor neurons. Disease duration was calculated from symptom onset to clinical assessment date, in months. Disability was scored using the amyotrophic lateral sclerosis functional rating scale revised (ALSFRS‐R)^24^, ^25^ and progression rate was calculated [i.e., (48 – ALSFRS‐R)/(disease duration from symptom onset in months)].^26^ Clinical staging was defined in each patient using the King's Clinical Staging Criteria System,^22^ which was shown to be closely linked to ALS anatomical spread.^27^ Briefly, this scale corresponds to the number of regions involved (bulbar, upper limb, lower limb, and respiratory or nutritional domains). From Stage 1 to Stage 3 there is an increase of the number of body regions presenting clinical signs of disease, on Stage 4 the respiratory or nutritional insufficiency require life‐saving intervention.

The study was conducted according to the principles set forth by the Declaration of Helsinki. The procedures involving human participants were reviewed and approved by the Ethical Committee of the IRCCS Neurological Institute “Carlo Besta.” All subjects gave written informed consent to participate in this study.

### Magnetic resonance imaging acquisition

**Figure 1:**
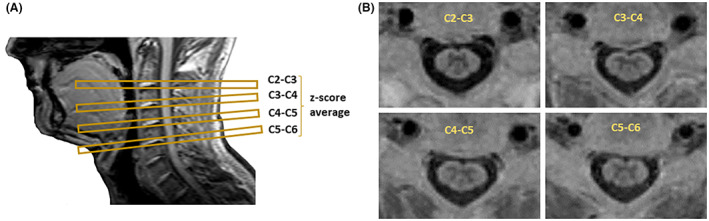
Example of PSIR magnetic resonance imaging acquisition. Panel A: axial 2D PSIR slices were acquired at four cervical segments (i.e., C2–C3, C3–C4, C4–C5, and C5–C6) perpendicular to the long axis of the cervical cord and centered at each intervertebral disc. Panel B: phase‐sensitive reconstructed PSIR images for each cervical segment.

Images were acquired using a 3T scanner (Achieva TX, Philips Healthcare) equipped with a 32‐channel coil. Whole‐brain 3D T1‐weighted images (Turbo Field Echo: repetition time (TR) = 8.4 ms, echo time (TE) = 3.9 ms, inversion time (TI) = 900 ms, flip angle = 8°, matrix = 240 × 240, voxel size = 1 × 1 × 1 mm^3^, 180 sagittal slices) as well as single slice axial 2D PSIR images (TR = 9.4 ms, TE = 4.6 ms, TI = 400 ms, flip angle = 10° axial in‐plane resolution = 0.78 × 0.78 mm^2^ slice thickness = 5 mm, matrix = 252 × 250, 3 averages, acquisition time: 2 min, magnitude and phase‐sensitive reconstructed images) at four cervical intervertebral disc segments (C2–C3, C3–C4, C4–C5, and C5–C6) were acquired as part of a more extensive magnetic resonance imaging protocol. The four axial 2D PSIR slices were acquired perpendicular to the long axis of the cervical cord and centered at each intervertebral disc (Fig. 1).

### Structural magnetic resonance imaging data analyses

For all subjects, TCA and GMA were estimated at the four different cervical segments from the phase‐sensitive reconstructed PSIR images using JIM software (http://www.xinapse.com). A single expert operator (N.P.) performed the measurements, blinded to the demographic and clinical characteristics of the cohort. TCA was measured semi‐automatically using the JIM “cord finder” tool with fixed settings, while GMA was manually computed, as previously reported.^18^, ^20^, ^21^ High intra‐ and inter‐rater reliability of measurements was shown.

#### Normalization of data for inter‐subject variability

V‐scale (representing the relative scaling of each participant's skull to the standard MNI152 space skull) and the product of the maximum anterior–posterior and lateral diameters of the spinal canal at the C2–C3 segment (i.e., surrogate of the canal area) were demonstrated to be the most effective normalization metrics to reduce the inter‐subject variability of cervical cord areas.^28^, ^29^

For each subject, V‐scale was obtained processing 3D‐T1 weighted images with SIENAX^30^ and surrogate of the canal area was measured on the PSIR images.

For each subject and cervical segment, TCA and GMA were normalized for inter‐subject variability using V‐scale and surrogate of the canal area following Model 3 of Ref. [28]. The (normalized) WMA was computed as the difference between the normalized TCA and GMA. When working segment by segment, we will refer to normalized areas in all the following sections of the manuscript unless otherwise specified.

To have a single value to represent the severity of SC atrophy in a subject we finally computed a z‐score average for the four cervical segments to take into account inter‐segment variability. For TCA, GMA, and WMA, z‐score was computed for each cervical segment for each subject according to the formula:

$$z-{\text{score}}^{i}=\left[{A^{i}}_{N–}\;\text{mean}\left({A^{\text{CTRLs}}}_{N}\right)\right]/\text{stdev}\left({A^{\text{CTRLs}}}_{N}\right),$$

where A^i^_N_ are the normalized TCA, GMA, and WMA for subject i, and mean (A^CTRLs^_N_) and stdev (A^CTRLs^_N_) are, respectively, their mean values and standard deviations for the group of healthy participants.

We computed the mean of the z‐score rather than the mean of the area because areas at the different cervical segments were different from each other and, by doing so, we would have weighted more the segments with bigger areas.

### Statistical analyses

#### Evaluation of SC atrophy across King's stages

Statistical analyses were performed using R software 4.0.3. Normal distribution of data was tested employing the Shapiro–Wilk test. The percentage reduction in TCA, WMA, and GMA of patients versus healthy participants was obtained for descriptive purposes. All the following statistical tests were separately applied to TCA, GMA, and WMA at the separate cervical segments and to the z‐score average for each tissue (henceforth SC metrics). Wilcoxon test was applied to SC metrics to assess differences between ALS patients and healthy participants. To assess if upper and lower motor neuron dominant phenotype had different SC atrophy, the Kruskal–Wallis test was applied to SC metrics among healthy participants and ALS patients grouped for upper motor neuron dominant versus lower motor neuron dominant. To assess if the level of the spinal cord involved at the time of MRI acquisition played a role in SC atrophy, the Kruskal–Wallis test was applied to SC metrics among healthy participants and ALS patients divided in three groups (i.e., cervical, lumbar and multi‐level involvement). We repeated this latter analysis restricting the entire cohort to the 31 spinal ‐ lower motor neuron dominant patients to verify if these patients had a different pattern of gray and white matter atrophy at the cervical level when only upper limbs were clinically affected. For this analysis, we, therefore, compared lower motor neuron dominant patients with some degree of upper limb involvement with lower motor neuron dominant patients with lower limb impairment only. Post‐hoc tests (Dunn's Kruskal–Wallis multiple comparisons) were used to assess between‐groups differences when the results for the Kruskal–Wallis tests were significant.

To assess whether there was a trend of reduced SC metrics as the King's stage increased, Jonckheere–Terpstra test (*p* < 0.05 with 1000 permutations) for ordered differences among groups was applied. Participants were categorized into four ordered groups based on King's stage independently from the phenotype (i.e., ordinal independent variable): healthy participants, ALS patients with King's stage 1, King's stage 2, and King's stage 3.

To assess the between‐group differences among healthy participants and ALS patients at the different King's stages, the Kruskal–Wallis test was applied to SC metrics to test for differences among healthy participants, ALS patients with King's stage 1, and ALS patients with King's stages> = 2. For significant tests, Dunn's Kruskal–Wallis multiple comparisons were used to assess between‐group differences. ALS patients with King's stage 2 and King's stage 3 were grouped to highlight the difference between the more (ALS patients with King's stages> = 2 participants) and less (ALS patients with King's stage 1) compromised patients.

Results were considered significant at *p* < 0.05 corrected for multiple comparisons (i.e., Holm‐Bonferroni method).

To assess the relationship between SC metrics in patients and the ALSFRS‐R score and disease duration from symptom onset (hereafter “linear regression model disease duration”), linear regression models (*p* < 0.05) were separately computed.

#### Estimation of time onset for SC atrophy

Since the motor neurons of anterior horns are the earliest foci^31^ affected by the pathological processes underlying ALS, we estimated the potential time of the onset of SC gray matter atrophy before the clinical manifestation of the disease, employing a linear disease progression model.^10^

To this aim, we applied the following steps:

- We identified if there was at least a SC segment where GMA was significantly correlated with disease duration (using the “linear regression model disease duration”);
- For the segments identified with step (i), we estimated the interquartile range (i.e., minimum and maximum of range) around the 25th percentile of the GMA distribution in the group of healthy participants using bootstrap test (1000 iterations). We arbitrarily defined this range as the interval in which the SC can be considered early atrophic;
- A straight line (i.e., regression line) was computed for each patient *i* using the slope coefficient of “linear regression model disease duration”.
- For each patient *i*, we identified the corresponding time‐points at which the regression line of the “linear regression model disease duration” intersected the maximum and the minimum of interquartile range in the group of healthy participants. For all patients, mean values of time‐points intersecting the maximum and the minimum of interquartile range, respectively, were computed allowing to estimate the possible time onset range of SC area atrophy before the clinical symptoms.

## Results

Four ALS patients were excluded from the initial cohort due to motion artifacts in the 2D PSIR acquisitions. Our final cohort included 44 early‐onset ALS patients with slow disease progression according to Kimura et al.^26^ Clinical and demographic data are summarized in Table 1. Due to low gray/white matter contrast in the 2D PSIR acquisitions, we could not assess the GMA and WMA at some segments in three patients [1 (upper motor neuron, ALS patients with King's stage 1) at C3–C4, 1 (lower motor neuron, ALS patients with King's stage 1) at C4–C5, 1 (lower motor neuron, ALS patients with King's stage 1) at all segments]: they were dealt as missing data in the analyses. No significant differences between ALS patients and healthy participants were reported for age [H (1) = 253.5; *p* = 0.06] and gender [*χ* ^2^(1, *N* = 61) = 0.73; *p* = 0.39]. Patients predominantly showed slow disease progression (median = 0.29 ALSFRS‐R points decline/month).^32^ The site of onset was evenly distributed among the King's stages, with the largest percentage of patients presenting lower motor neuron predominant phenotype (71%) and all limbs involvement (59%).

**Table 1:** Demographic and clinical characteristics of study participants (median and interquartile range).

	ALS (*n* = 44)				CTRL (*n* = 17)
	K1 (*n* = 25)^a^	K2 (*n* = 14)	K3 (*n* = 5)	All (*n* = 44)	
*Demographic data*					
Age (years)	64 (52–69)	64 (52.8–71.8)	70 (59–72)	64.5 (52–71)	56 (49–60.8)
Male/female	11/14	7/7	2/3	20/24	5/12
*Clinical data*					
Site of onset [*n* (*%*)]					
SC level involved at MRI [*n*]					
Bulbar	4 (16)	1 (7)	0 (0)	5 (11)	–
Bulbar	4	0	0	4	–
Bulbar + cervical	0	1	0	1	–
Spinal – UMN dominant	5 (20)	2 (14)	1 (20)	8 (18)	–
Bulbar	2	0	0	2	–
Lumbar	3	0	0	3	–
Bulbar + lumbar	0	1	0	1	–
Cervical + lumbar	0	1	0	1	–
Bulbar + cervical + lumbar	0	0	1	1	–
Spinal – LMN dominant	16 (64)	11 (79)	4 (80)	31 (71)	–
Cervical	7	1	0	8	–
Lumbar	9	0	0	9	–
Cervical + lumbar	0	10	0	10	–
Bulbar + cervical + lumbar	0	0	4	4	–
ALSFRS‐R (points)	44 (43–46)	42.5 (39.5–44.8)	36 (36–37)	43.5 (41–46)	NA
Disease duration (months)	13 (9–26)	14 (8.5–23)	32 (10–35)	14 (9–27)	NA
Disease progression rate (points/months)	0.27 (0.1–0.44)	0.28 (0.21–0.63)	0.38 (0.29–1.1)	0.29 (0.12–0.53)	NA

ALS patients are grouped according to site of onset, and further stratified by spinal cord (SC) levels involved at the time of MRI acquisition. Disease duration indicates the time interval from the onset of symptoms and the date of MRI scan.

ALS, amyotrophic lateral sclerosis patients; K1, King's stage 1; K2, King's stage 2; K3, King's stage 3; CTRL, healthy participants; LMN, lower motor neuron; mo, months; NA, not applicable; UMN, upper motor neuron.

^a^ Due to low gray/white matter contrast in the 2D PSIR acquisitions, GMA and WMA was not assessed in three patients at some segments: 1 (UMN, K1) at C3–C4, 1 (LMN, K1) at C4–C5, 1 (LMN, K1) at all segments.

### Evaluation of SC atrophy across King's stages

The effectiveness of normalization on TCA, GMA, and WMA metrics using V‐scale and surrogate of the canal area is reported in Table 2. On the whole cohort, the average standard deviation reduction at the 4 cervical segments was 10.33% for TCA and 6.28% for GMA. Considering the healthy participants only, the average standard deviation reduction at the 4 cervical segments was 15.57% for TCA and 14.09% for GMA.

**Table 2:** The normalization effectiveness comparing the standard deviation (stdev) of the 4 cervical levels before (raw data) and after (norm. data) normalization on the whole cohort and on the group of healthy participants only.

	Cervical segments			
	C2–C3	C3–C4	C4–C5	C5–C6
Whole cohort				
TCA				
stdev of raw data	8.656	10.437	10.950	8.623
stdev of norm. data	7.901	9.511	9.659	7.587
% reduction	8.63%	8.87%	11.79%	12.01%
GMA				
stdev of raw data	1.938	2.860	3.039	2.972
stdev of norm. data	1.875	2.681	2.768	2.772
% reduction	3.24%	6.26%	8.91%	6.70%
Healthy participants				
TCA				
stdev of raw data	7.509	7.806	9.713	7.434
stdev of norm. data	6.265	6.597	8.053	6.458
% reduction	16.57%	15.49%	17.09%	13.13%
GMA				
stdev of raw data	1.349	1.660	2.354	2.414
stdev of norm. data	1.026	1.493	1.938	2.301
% reduction	23.93%	10.10%	17.68%	4.66%

GMA, gray matter area; TCA, total cross‐sectional area.

ALS patients showed a significant reduction in both GMA (9.7–14.3%) and WMA (4.5–11.3%) versus healthy participants in all SC metrics (Wilcoxon test: *p* ≤ 0.005; Table 3).

**Table 3:** SC area comparison between healthy participants and ALS patients according to King's clinical stage.

	Percentage of atrophy compared to Healthy p. (*%)*				Trend (Jonckheere–Terpstra test)	ALS versus Healthy p.		King1 versus King ≥ 2 versus Healthy p.		King1 versus Healthy p.	King ≥ 2 versus Healthy p.	King1 versus King ≥ 2
	All ALS	King1	King2	King3	*p‐*value	*W*	*p‐*value	*χ2*	*p‐*value	*p‐*value	*p‐*value	*p‐*value
TCA												
z‐score average	–	–	–	–	**<0.001**	621	**<0.001**	23.53	**<0.001**	**0.014**	**<0.001**	**0.008**
C2–C3	8.24	4.1	11.96	13.55	**<0.001**	581	**0.001**	15.58	**<0.001**	**0.049**	**<0.001**	**0.034**
C3–C4	10.04	6.11	12.07	15.98	**<0.001**	625	**<0.001**	20.72	**<0.001**	**0.008**	**<0.001**	**0.036**
C4–C5	10.91	6.19	13.47	17.45	**<0.001**	619	**<0.001**	23.36	**<0.001**	**0.016**	**<0.001**	**0.008**
C5–C6	8.24	3.66	13.54	9.89	**<0.001**	588	**<0.001**	25.00	**<0.001**	0.107	**<0.001**	**<0.001**
GMA												
z‐score average	–	–	–	–	**<0.001**	635	**<0.001**	27.11	**<0.001**	**0.007**	**<0.001**	**0.006**
C2–C3	9.66	7.38	11.18	11.84	**<0.001**	558	**0.001**	13.09	**0.001**	0.054	**0.001**	0.077
C3–C4	14.26	10.86	17.28	21.12	**<0.001**	635	**<0.001**	26.20	**<0.001**	**0.002**	**<0.001**	**0.033**
C4–C5	13.63	9.97	16.86	22.46	**<0.001**	616	**<0.001**	24.11	**<0.001**	**0.006**	**<0.001**	**0.021**
C5–C6	12.9	5.79	20.34	18.12	**<0.001**	558	**0.001**	23.66	**<0.001**	0.204	**<0.001**	**<0.001**
WMA												
z‐score average	–	–	–	–	**<0.001**	574	**<0.001**	20.78	**<0.001**	0.073	**<0.001**	**0.004**
C2–C3	9.07	3.28	13.52	15.59	**<0.001**	536	**0.005**	14.34	**<0.001**	0.151	**0.001**	**0.016**
C3–C4	9.73	6.17	13.66	15.86	**<0.001**	542	**0.002**	16.19	**<0.001**	0.098	**<0.001**	**0.015**
C4–C5	11.26	6.1	14.64	16.64	**<0.001**	560	**<0.001**	23.45	**<0.001**	0.123	**<0.001**	**0.001**
C5–C6	4.45	2.47	10.82	10.45	**<0.001**	554	**0.002**	18.63	**<0.001**	0.132	**<0.001**	**0.004**

The percentage reduction of SC metrics in patients in comparison to healthy participants according to King's clinical stage is reported. The Jonckheere–Terpstra test assessed the trend of SC atrophy according to ordered differences among classes, that is, healthy participants, ALS patients with King's staging system score = 1 (King1), ALS patients with King's staging system score = 2 (King2), and ALS patients with King's staging system score = 3 (King3). Wilcoxon tests were used to assess the differences between ALS patients and healthy participants while Kruskal–Wallis tests were used to analyze between‐groups differences among healthy participants, ALS patients with King's staging system score = 1, and ALS patients with King's staging system score> = 2. Results were corrected for multiple comparisons. Significant results are highlighted in bold.

ALS, amyotrophic lateral sclerosis patients; GMA, gray matter area; Healthy p., healthy participants; SC, spinal cord; TCA, total cord area; WMA, white matter area.

Significant between‐group differences among healthy participants, lower motor neuron, and upper motor neuron dominant patients were detected for all SC metrics (Kruskal–Wallis test: *p* ≤ 0.026), while no significant difference was observed between upper and lower motor neuron dominant patients (Table 4). Similarly, significant between‐group differences among healthy participants and patients grouped for the level of the spinal cord involved at the time of MRI acquisition were detected (Kruskal–Wallis test: *p* ≤ 0.021, see Supplementary materials). In post‐hoc tests, no differences were identified between cervical and lumbar patients when considering the entire cohort (Table S1, Fig. S1), while a significant reduction in GMA of the C5–C6 segment was detected in lower motor neuron dominant patients with some degree of upper limb involvement when compared to lower motor neuron dominant patients with lower limb involvement only (Table S2, Fig. S2).

**Table 4:** SC metrics between healthy participants and ALS patients according to site of onset and lower and upper motor neuron dominant involvement.

	Percentage of atrophy compared to Healthy p. (*%)*				UMN versus LMN versus Healthy p.		UMN versus Healthy p.	LMN versus Healthy p.	UMN versus LMN
	All ALS	UMN	LMN	Bulbar	*χ2*	*p‐*value	*p‐*value	*p‐*value	*p‐*value
TCA									
z‐score average	–	–	–	–	15.972	**<0.001**	**0.03**	**<0.001**	0.616
C2–C3	9.16	9.28	9.36	7.76	11.287	**0.004**	**0.039**	**0.003**	0.948
C3–C4	11.62	10.97	12.31	8.41	16.803	**<0.001**	**0.041**	**<0.001**	0.474
C4–C5	11.98	9.00	12.68	12.40	14.896	**0.001**	**0.042**	**<0.001**	0.593
C5–C6	8.83	8.08	9.34	6.89	11.934	**0.003**	**0.044**	**0.002**	0.833
GMA									
z‐score average	–	–	–	–	22.051	**<0.001**	**0.016**	**<0.001**	0.42
C2–C3	7.28	6.76	8.14	2.96	11.966	**0.003**	0.055	**0.002**	0.732
C3–C4	13.68	11.10	15.39	7.02	23.607	**<0.001**	**0.017**	**<0.001**	0.436
C4–C5	13.19	9.23	14.42	12.39	20.214	**<0.001**	**0.034**	**<0.001**	0.323
C5–C6	11.24	10.75	12.29	5.71	10.554	**0.005**	0.06	**0.005**	0.833
WMA									
z‐score average	–	–	–	–	10.973	**0.004**	0.067	**0.003**	0.745
C2–C3	9.34	10.15	9.11	9.44	7.314	**0.026**	0.069	**0.034**	0.826
C3–C4	9.39	5.28	10.42	8.95	10.124	**0.006**	0.392	**0.005**	0.276
C4–C5	10.40	8.91	10.47	12.40	10.084	**0.006**	**0.045**	**0.007**	0.997
C5–C6	7.59	7.04	7.77	7.35	9.156	**0.01**	0.074	**0.010**	0.902

The percentage reduction of SC metrics in spinal (upper and lower motor neuron dominant) and bulbar patients in comparison to healthy participants (%) is reported. Kruskal–Wallis tests were used to assess the between‐groups differences among healthy participants, upper and lower motor neuron dominant involvement patients. Bulbar patients were not included in this analysis due to the small number of cases (i.e., *n* = 5). Results were corrected for multiple comparisons. Significant results are highlighted in bold.

ALS, amyotrophic lateral sclerosis patients; Healthy p., healthy participants; LMN, lower motor neuron dominant involvement; UMN, upper motor neuron dominant involvement.

**Figure 2:**
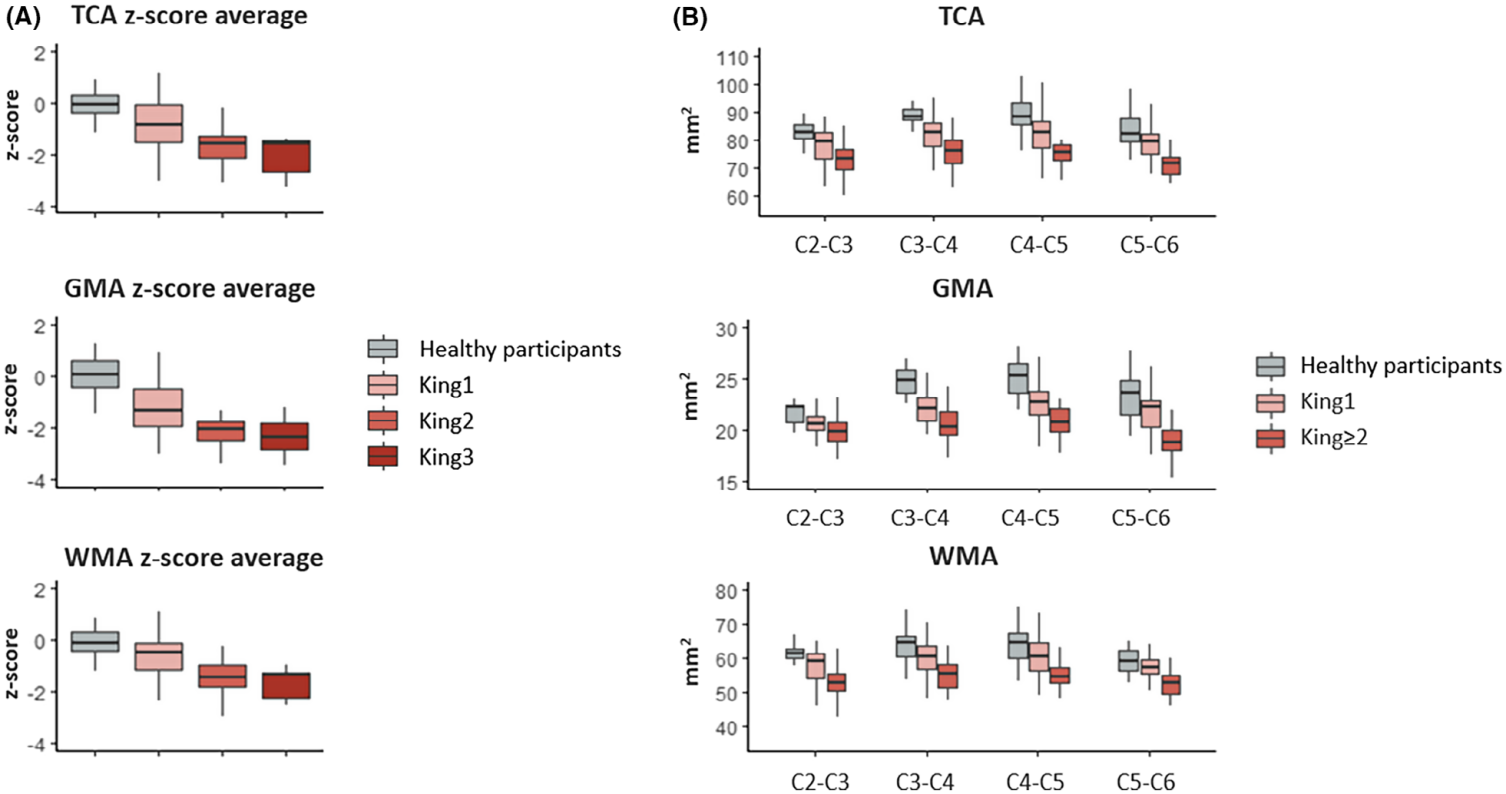
SC metrics for different King's clinical stags and for the different segments. Panel A: boxplot of TCA, GMA and WMA as z‐score average among healthy participants and ALS patients according to King's clinical stage [i.e., ALS patients with King's staging system score = 1 (King1), ALS patients with King's staging system score = 2 (King2), and ALS patients with King's staging system score = 3 (King3)]. Panel B: boxplot of TCA, GMA and WMA for each cervical intervertebral disc segment (i.e., C2–C3, C3–C4, C4–C5, and C5–C6) among groups. In this plot, King2 and King3 patients were grouped to highlight the difference between the more (ALS patients with King's staging system score ≥ 2) and less (ALS patients with King's staging system score = 1) compromised patients.

The SC metrics among the ordered King's stages showed a significant trend of increasing atrophy from healthy participants to patients with King's stage 3 in TCA, GMA, and WMA at each segment and z‐score average (Jonckheere–Terpstra test: *p* ≤ 0.001; Table 3; Fig. 2A).

Significant between‐group differences among healthy participants and ALS patients grouped by King's stages were detected for all SC metrics (Kruskal–Wallis test: *p* ≤ 0.001; Table 3). In post‐hoc tests, ALS patients with King's stage 1 compared to healthy participants showed a significant reduction of TCA and GMA, particularly in z‐score average (TCA: *p* = 0.014; GMA: *p* = 0.007) (Fig. 2A). This atrophy was mainly localized from C2–C3 to C4–C5 segments for TCA and C3–C4 and C4–C5 segments for GMA. ALS patients with King's stage ≥ 2 compared to healthy participants presented significant atrophy for all segments extending not only to the gray matter but also to the white matter. ALS patients with King's stage 1 differed from ALS patients with King's stage ≥ 2 prevalently in WMA, but also in GMA as z‐score average, although to a lesser extent (Table 3, Fig. 2B). The marked atrophy was mainly localized in the GMA from C3–C4 to C5–C6 segments and the WMA of C2–C3 to C5–C6 segments, increasing caudally (Table 3, Fig. 2B).

Linear regression showed a strong positive association between TCA, GMA, and WMA for all segments and ALSFRS‐R score, except for the C5–C6 segment for GMA (Table 5). The association was more marked in white matter than gray matter for all segments. Moreover, we detected a negative association between TCA and WMA as z‐score average (Table 5) and disease duration. This relationship was also present in TCA for all 4 segments, in GMA of C3–C4 segment, and in WMA from C3–C4 to C5–C6 segments. C3–C4 was the segment at which GMA and WMA were mainly correlated with disease duration.

**Table 5:** Linear regression of SC metrics with ALSFRS‐R and disease duration and estimation of time onset for SC atrophy in ALS patients.

	ALSFRS‐R		Disease duration	
	*R* ^2^	*p‐*value	*R* ^2^	*p‐*value
TCA				
z‐score average	0.22	**0.001**	0.160	**0.007**
C2–C3	0.24	**0.001**	0.114	**0.025**
C3–C4	0.124	**0.019**	0.141	**0.012**
C4–C5	0.256	**<0.001**	0.192	**0.003**
C5–C6	0.152	**0.009**	0.102	**0.034**
GMA				
z‐score average	0.231	**0.001**	0.059	0.116
C2–C3	0.223	**0.001**	0.028	0.284
C3–C4	0.138	**0.016**	0.123	0.023 (*)
C4–C5	0.172	**0.006**	0.028	0.288
C5–C6	0.089	0.053	0.015	0.430
WMA				
z‐score average	0.271	**<0.001**	0.120	**0.023**
C2–C3	0.258	**0.001**	0.082	0.062
C3–C4	0.267	**<0.001**	0.127	**0.021**
C4–C5	0.317	**<0.001**	0.112	**0.030**
C5–C6	0.163	**0.007**	0.097	**0.042**

Linear regression of SC metrics with ALSFRS‐R and disease duration (linear regression model disease duration). (*) Estimation of time onset of SC GMA atrophy before the clinical manifestation of disease symptomatology using “linear regression model disease duration”: interquartile range as the minimum and the maximum of the distribution around the 25th percentile in GMA of the group of healthy participants = 22.9/23.7 mm^2^; angular coefficient of “linear regression model disease duration” = −0.061; estimated time onset for SC area atrophy in relation to the clinical symptoms obtained as the points at which the regression line intersected the interquartile range (negative values indicate time before the clinical symptoms) = −20/−7 months. Significant results are highlighted in bold.

ALSFRS‐R, amyotrophic lateral sclerosis functional rating scale.

### Estimation of time onset for SC atrophy

**Figure 3:**
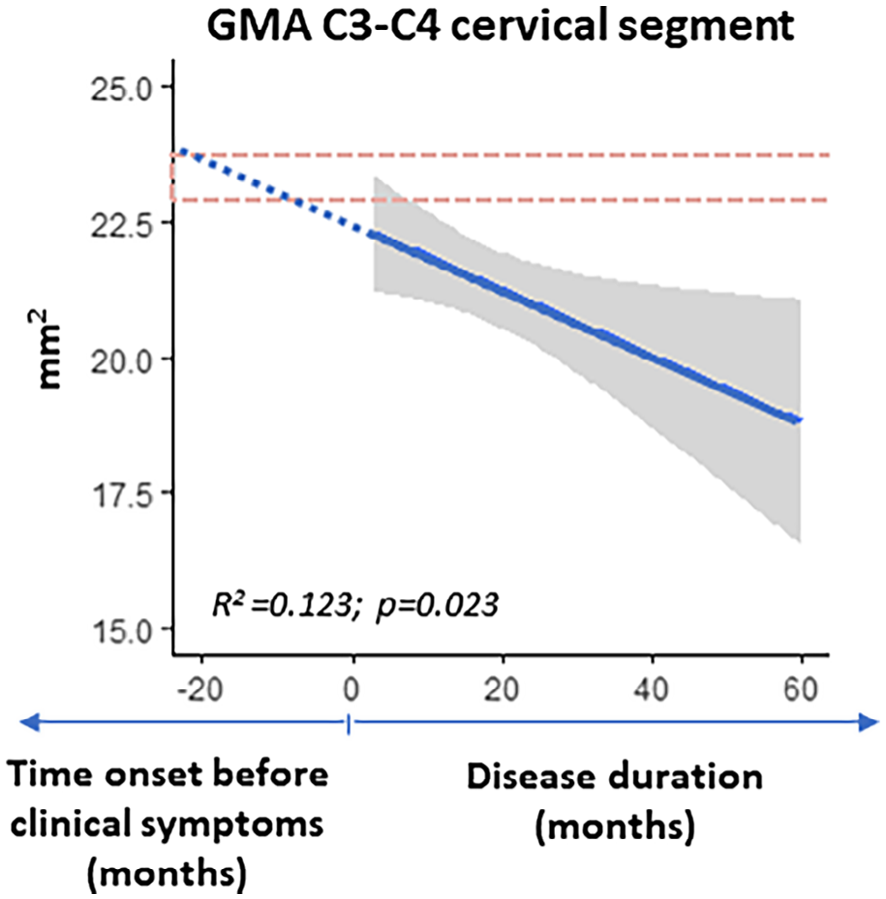
Linear regression model inferring the potential onset of cervical gray matter atrophy. In the cohort under investigation, GMA at the cervical segment C3–C4 decreased significantly as disease duration increased. For descriptive purposes of method: the horizontal dashed lines represent the maximum and the minimum of interquartile range of GMA in the group of healthy participants; blue line represents the significant linear regression with disease duration (linear regression model disease duration) for GMA at the cervical segment C3–C4, used to estimate possible time of onset of SC atrophy before clinical manifestation of disease symptoms (dashed part of the blue line); gray region represents standard error of the linear regression estimation.

The linear disease progression model was computed for the GMA of the segment C3–C4, which presented a significant correlation with disease duration according to the “linear regression model disease duration” (Table 5). According to our model, the atrophy in this segment is likely to appear from 7 to 20 months before the clinical onset of the disease (GMA in C3–C4: healthy participants interquartile range = 22.9/23.7 mm^2^; slope coefficient of “linear regression model disease duration” = −0.061; estimation range of time onset = −20/−7 months) (Fig. 3).

## Discussion

Our cross‐sectional study investigated the spatial patterns of SC atrophy in a cohort of ALS patients stratified by the King's clinical stage. Using a reliable segmentation method on 2D PSIR images, we were able to estimate TCA, GMA, and WMA at four cervical intervertebral disc segments (C2 to C6). The main findings of the study are: (i) TCA, GMA, and WMA in ALS patients were significantly reduced compared to healthy participants at all four cervical segments; (ii) the first evidence of significant cervical SC alteration was identified in gray matter for patients at King's stage 1 in comparison to healthy participants, whereas, in patients at King's stage ≥ 2, both gray and white matter were significantly reduced when compared to healthy participants and patients at King's stage 1; (iii) TCA, GMA, and WMA at all segments correlated with clinical severity measured by ALSFRS‐R; (iv) the cervical segment affected first by gray matter atrophy was the C3–C4 level, with an estimated onset time range of SC area atrophy approximately 7–20 months before symptom onset according to our model. Interestingly, the differences in SC atrophy related to the clinical stage of the disease seemed independent from the upper and lower motor neuron dominant involvement of these patients as well as the level of spinal cord involvement at the MRI acquisition, as we reported no differences between these ALS phenotypes.

As reported in previous studies, we found that TCA in ALS patients was significantly reduced in comparison to healthy participants.^5^, ^6^, ^7^, ^8^, ^10^, ^11^, ^12^, ^18^ So far, only three studies have separately assessed SC GMA and WMA in patients with ALS using magnetic resonance imaging.^8^, ^17^, ^18^ In agreement with our findings, these studies reported significant atrophy in both gray^8^, ^17^, ^18^ and white^17^, ^18^ matter of the cervical SC in small samples of ALS and motor neuron disease patients, showing in particular that GMA more than TCA was a sensitive marker of atrophy in ALS.^8^

In addition, our cross‐sectional study showed that SC atrophy followed a trend of increasing atrophy along the investigated cervical portion of the SC as King's clinical stage increases, with an earlier involvement of gray and white matter. In detail, when compared with healthy participants, patients with involvement of a single anatomical region (i.e., patients with King's stage 1) showed atrophy limited to the gray matter, whereas those with multiple central nervous system regions involved (i.e., patients with King's stage 2 and 3) had atrophy involving not only the gray matter but also the white matter. Previous SC studies in postmortem patients showed early extensive degeneration of gray matter oligodendrocytes.^31^ Specifically, in the early stages of disease evolution in patients, pTDP‐43 inclusions were present in the SC gray matter oligodendroglia and absent in the SC white matter oligodendroglia. By contrast, in more advanced stages of the disease with a greater burden of neuronal TDP‐43 lesions, pTDP‐43 inclusions were widespread in both gray and white matter oligodendroglia.^31^

SC atrophy affected the various cervical segments differently depending on the disease stage, but independently from clinical phenotype or motor neuron dominant involvement. We observed prominent atrophy at the C3–C4 segment followed by the C4–C5 segment in patients in King's stage 1, and of the more caudal segments (i.e., C4–C5 and C5–C6) at more advanced King's stages. This observation is consistent with the progression of pathology to the caudal portion of the cervical cord reported in previous longitudinal studies.^10^, ^11^ Interestingly, we detected a reduction of GMA of the C5–C6 segment in lower motor neuron dominant patients with some degree of clinical upper limb involvement when compared to patients without upper limb involvement. These results seem to support the hypothesis of a different pattern of gray and white matter atrophy in the cervical SC of patients with upper limb versus lower limb involvement, but have to be confirmed in a larger cohort.^16^

Also in line with previous studies, in our ALS patients TCA was associated with ALSFRS‐R and disease duration.^6^, ^7^, ^8^, ^10^, ^11^, ^16^ Furthermore, we showed that there was a strong positive correlation with ALSFRS‐R in WMA as well as GMA, although WMA showed the higher correlation.

In our cohort, the C3–C4 cervical segment appeared to be a key region in the earliest phases of disease evolution. It showed early gray matter alterations and a strong correlation with disease duration in both gray and white matter. Notably, assuming a linear progression of pathology our model estimated that SC gray matter atrophy in the C3–C4 segment is likely to start 7–20 months before clinical symptoms become apparent. The crucial role that this segment played in the early phase and disease progression of ALS in our study seems consistent with previous studies in early ALS patients that showed alterations in SC area localized at C3 and/or C4 segments both cross‐sectionally, associated with lower survival index,^8^, ^14^ and longitudinally.^10^, ^11^

Altogether our new findings corroborate the potential role of SC gray and white matter area quantification as a valuable additional tool to quantify the spread of the disease in vivo and to monitor disease progression in future longitudinal research studies.

The investigation of the thoracic and lumbar portion of the SC would complete the scenario. PSIR and T2*‐weighted MRI protocols have been successfully applied to the thoracic and lumbar SC of multiple sclerosis patients and healthy controls.^20^, ^33^, ^34^

However, adding the exploration of thoracic and lumbar segments to the MRI session would have required longer acquisition times and introduced more discomfort for patients. We have decided to focus our study on the cervical SC, but a future study including other portions of the SC is warranted, in particular to validate the findings of a recent study on 15 ALS patients and 17 healthy participants that found evidence of TCA reduction around the cervical but not the thoracolumbar enlargement of the cord in the ALS participants.^13^

Some limitations of our study should be noted.

First, in this study we used a manual segmentation of GMA. However, it was shown that the acquisition and processing methods used here have high intra‐ and inter‐operator reliability.^18^, ^20^, ^21^ Free‐access software that can provide automated segmentation of SC tissues is nowadays available, and its use has the potential to substantially improve SC atrophy analyses in terms of operator dependency and time. The Spinal Cord Toolbox (SCT)^35^ is undoubtedly the most complete and widely used software currently available in the field. While the automated methods included in the SCT provide reliable TCA estimates on MRI images of different resolution and contrast,^19^ the performance of the SCT and other available tools for GM segmentation has not been tested on images acquired with 2D PSIR protocols or at cervical segments distinct from C2 to C3.^36^, ^37^, ^38^ Training a Deep learning method on PSIR images acquired at multiple cervical segments is the most promising approach to develop an automated GM segmentation method that would make the results of the present study easily translatable to clinical practice.

Second, our results may be biased by the prevalence of lower motor neuron dominant patient and slow progressors. Nonetheless, patients were equally distributed among King's stages for upper and lower motor neuron dominant phenotype and the slow disease progression may have allowed us to closely staging the disease in gray and white matter.

Third, we assumed a linear model for disease progression in SC gray matter, that might overestimate the possible onset of SC atrophy. However, this assumption is consistent with prior longitudinal studies.^10^, ^11^ Further studies in early‐stage patients, longitudinally assessed, will be necessary to confirm the pattern of disease progression in gray matter and white matter along the cervical SC.

## Conclusions

Our findings support the hypothesis of a progressive spread of degeneration starting from gray to white matter cervical cord across King's clinical stages in ALS. The C3–C4 segment seems to be a key region of pathology in the early stage of the disease and we speculate that alterations in gray matter of this segment could appear up to 20 months before the clinical symptoms. In vivo measurement of SC gray matter areas using magnetic resonance imaging may be useful to track anatomical ALS disease spread from its earliest stages.

## Author Contributions

AN, EDB, SF, GL, MGB, and NP were involved in conception and design of the study. AN, EDB, SF, JPM, GD, EB, MC, AB, MS, SP, and NP were involved in acquisition, analysis, or interpretation of data. AN, EDB, SF, JPM, and NP were involved in drafting of the manuscript GD, EB, MC, AB, MS, SP, GL, and MGB were involved in critical revision of the manuscript for important intellectual content. AN, SF, JPM, and NP were involved in statistical analysis. GL was involved in obtained funding. GL, MGB, and NP were involved in supervision.

## Conflict of Interest

The authors declare that the research was conducted in the absence of any commercial or financial relationships that could be construed as a potential conflict of interest.

## Supporting information


**Table S1.** SC metrics between healthy participants and ALS patients according to the level of the spinal cord involved at the time of MRI acquisition.
**Table S2.** SC metrics between healthy participants and lower motor neuron dominant patients with upper and lower limb involvement.
**Figure S1.** SC metrics for patients grouped according to the level of the spinal cord involved at the time of MRI acquisition.
**Figure S2.** SC metrics for lower motor neuron dominant patients with upper and lower limb involvement at the time of MRI acquisition.

## Data Availability

The datasets presented in this article are not readily available because the files cannot be deposited in an accessible repository online in compliance with the regulations on the processing and dissemination of personal data in the health sector (application of the EU Regulation 2016/679 (GDPR) and the Privacy Italian Law as updated by Legislative Decree 101/2018). Requests to access the datasets should be directed to the corresponding author.

## References

[1] Van Es MA , Hardiman O , Chio A , et al. Amyotrophic lateral sclerosis. Lancet. 2017;390:2084‐2098. 28552366 10.1016/S0140-6736(17)31287-4

[2] Chiò A , Logroscino G , Traynor BJ , et al. Global epidemiology of amyotrophic lateral sclerosis: a systematic review of the published literature. Neuroepidemiology. 2013;41:118‐130. 23860588 10.1159/000351153 PMC4049265

[3] Wijesekera LC , Leigh PN . Amyotrophic lateral sclerosis. Orphanet J Rare Dis. 2009;4:1‐22. 19192301 10.1186/1750-1172-4-3 PMC2656493

[4] El Mendili MM , Querin G , Bede P , et al. Spinal cord imaging in amyotrophic lateral sclerosis: historical concepts – novel techniques. Front Neurol. 2019;10:1‐11. 31031688 10.3389/fneur.2019.00350 PMC6474186

[5] Cohen‐Adad J , El MMM , Morizot‐Koutlidis R , et al. Involvement of spinal sensory pathway in ALS and specificity of cord atrophy to lower motor neuron degeneration. Amyotroph Lateral Scler Front Degener. 2013;14:30‐38. 10.3109/17482968.2012.701308 22881412

[6] Branco LMT , De Albuquerque M , De Andrade HMT , et al. Spinal cord atrophy correlates with disease duration and severity in amyotrophic lateral sclerosis. Amyotroph Lateral Scler Front Degener. 2014;15:93‐97. 10.3109/21678421.2013.852589 24219347

[7] de Albuquerque M , Branco LMT , Rezende TJR , de Andrade HMT , Nucci A , França MC Jr . Longitudinal evaluation of cerebral and spinal cord damage in amyotrophic lateral sclerosis. NeuroImage Clin. 2017;14:269‐276. 28203530 10.1016/j.nicl.2017.01.024 PMC5294732

[8] Paquin ME , Mendili MME , Gros C , et al. Spinal cord gray matter atrophy in amyotrophic lateral sclerosis. Am J Neuroradiol. 2018;39:184‐192. 29122760 10.3174/ajnr.A5427 PMC7410702

[9] Grolez G , Kyheng M , Lopes R , et al. MRI of the cervical spinal cord predicts respiratory dysfunction in ALS. Sci Rep. 2018;8:6‐11. 29379040 10.1038/s41598-018-19938-2 PMC5789036

[10] van der Burgh HK , Westeneng HJ , Meier JM , et al. Cross‐sectional and longitudinal assessment of the upper cervical spinal cord in motor neuron disease. NeuroImage Clin. 2019;24:101984. 31499409 10.1016/j.nicl.2019.101984 PMC6734179

[11] Wimmer T , Schreiber F , Hensiek N , et al. The upper cervical spinal cord in ALS assessed by cross‐sectional and longitudinal 3T MRI. Sci Rep. 2020;10:1‐10. 32020025 10.1038/s41598-020-58687-z PMC7000761

[12] Pisharady PK , Eberly LE , Cheong I , et al. Tract‐specific analysis improves sensitivity of spinal cord diffusion MRI to cross‐sectional and longitudinal changes in amyotrophic lateral sclerosis. Commun Biol. 2020;3:1‐13. 32651439 10.1038/s42003-020-1093-z PMC7351722

[13] Barry RL , Torrado‐Carvajal A , Kirsch JE , et al. Selective atrophy of the cervical enlargement in whole spinal cord MRI of amyotrophic lateral sclerosis. NeuroImage Clin. 2022;36:103199. 36137496 10.1016/j.nicl.2022.103199 PMC9668597

[14] Querin G , El Mendili MM , Lenglet T , et al. Spinal cord multi‐parametric magnetic resonance imaging for survival prediction in amyotrophic lateral sclerosis. Eur J Neurol. 2017;24:1040‐1046. 28586096 10.1111/ene.13329

[15] Agosta F , Rocca MA , Valsasina P , et al. A longitudinal diffusion tensor MRI study of the cervical cord and brain in amyotrophic lateral sclerosis patients. J Neurol Neurosurg Psychiatry. 2009;80:53‐55. 18931009 10.1136/jnnp.2008.154252

[16] El Mendili MM , Cohen‐Adad J , Pelegrini‐Issac M , et al. Multi‐parametric spinal cord MRI as potential progression marker in amyotrophic lateral sclerosis. PLoS One. 2014;9:1‐7. 10.1371/journal.pone.0095516 PMC3995720 24755826

[17] Rasoanandrianina H , Grapperon AM , Taso M , et al. Region‐specific impairment of the cervical spinal cord (SC) in amyotrophic lateral sclerosis: a preliminary study using SC templates and quantitative MRI (diffusion tensor imaging/inhomogeneous magnetization transfer). NMR Biomed. 2017;30:1‐13. 10.1002/nbm.3801 28926131

[18] Olney NT , Bischof A , Rosen H , et al. Measurement of spinal cord atrophy using phase sensitive inversion recovery (PSIR) imaging in motor neuron disease. PLoS One. 2018;13:1‐15. 10.1371/journal.pone.0208255 PMC6264489 30496320

[19] Papinutto N , Henry RG . Evaluation of intra‐ and Interscanner reliability of MRI protocols for spinal cord gray matter and Total cross‐sectional area measurements. J Magn Reson Imaging. 2019;49:1078‐1090. 30198209 10.1002/jmri.26269 PMC6620602

[20] Papinutto N , Schlaeger R , Panara V , et al. 2D phase sensitive inversion recovery imaging to measure in‐ vivo spinal cord gray and white matter areas in clinically feasible acquisition times. J Magn Reson Imaging. 2015;42:698‐708. 25483607 10.1002/jmri.24819 PMC5953416

[21] Schlaeger R , Papinutto N , Panara V , et al. Spinal cord gray matter atrophy correlates with multiple sclerosis disability. Ann Neurol. 2014;76:568‐580. 25087920 10.1002/ana.24241 PMC5316412

[22] Roche JC , Rojas‐Garcia R , Scott KM , et al. A proposed staging system for amyotrophic lateral sclerosis. Brain. 2012;135:847‐852. 22271664 10.1093/brain/awr351 PMC3286327

[23] Brooks BR , Miller RG , Swash M , Munsat TL . El Escorial revisited: revised criteria for the diagnosis of amyotrophic lateral sclerosis. Amyotroph Lateral Scler. 2000;1:293‐299. 10.1080/146608200300079536 11464847

[24] Cedarbaum JM , Stambler N , Malta E , et al. The ALSFRS‐R: a revised ALS functional rating scale that incorporates assessments of respiratory function. J Neurol Sci. 1999;169:13‐21. 10540002 10.1016/s0022-510x(99)00210-5

[25] Bakker LA , Schröder CD , Tan HHG , et al. Development and assessment of the inter‐rater and intra‐rater reproducibility of a self‐administration version of the ALSFRS‐R. J Neurol Neurosurg Psychiatry. 2020;91:75‐81. 31558653 10.1136/jnnp-2019-321138

[26] Kimura F , Fujimura C , Ishida S , et al. Progression rate of ALSFRS‐R at time of diagnosis predicts survival time in ALS. Neurology. 2006;38:265‐267. 10.1212/01.wnl.0000194316.91908.8a 16434671

[27] Fang T , Al Khleifat A , Stahl DR , et al. Comparison of the King's and MiToS staging systems for ALS. Amyotroph Lateral Scler Front Degener. 2017;18:227‐232. 10.1080/21678421.2016.1265565 PMC5425622 28054828

[28] Papinutto N , Asteggiano C , Bischof A , et al. Intersubject variability and normalization strategies for spinal cord total cross‐sectional and gray matter areas. J Neuroimaging. 2020;30:110‐118. 31571307 10.1111/jon.12666 PMC7153493

[29] Papinutto N , Cordano C , Asteggiano C , et al. MRI measurement of upper cervical spinal cord cross‐sectional area in children. J Neuroimaging. 2020;30:598‐602. 32639671 10.1111/jon.12758 PMC7530010

[30] Smith SM , Zhang Y , Jenkinson M , et al. Accurate, robust, and automated longitudinal and cross‐sectional brain change analysis. Neuroimage. 2002;17:479‐489. 12482100 10.1006/nimg.2002.1040

[31] Brettschneider J , Arai K , Del Tredici K , et al. TDP‐43 pathology and neuronal loss in amyotrophic lateral sclerosis spinal cord. Acta Neuropathol. 2014;128:423‐437. 24916269 10.1007/s00401-014-1299-6 PMC4384652

[32] Labra J , Menon P , Byth K , Morrison S , Vucic S . Rate of disease progression: a prognostic biomarker in ALS. J Neurol Neurosurg Psychiatry. 2016;87:628‐632. 26152368 10.1136/jnnp-2015-310998

[33] Schlaeger R , Papinutto N , Zhu AH , et al. Association between thoracic spinal cord gray matter atrophy and disability in multiple sclerosis. JAMA Neurol. 2015;72:897‐904. 26053119 10.1001/jamaneurol.2015.0993 PMC6002864

[34] Yiannakas MC , Kakar P , Hoy LR , et al. The use of the lumbosacral enlargement as an intrinsic imaging biomarker: feasibility of grey matter and white matter cross‐sectional area measurements using MRI at 3T. PLoS One. 9. Epub ahead of print 2014. doi:10.1371/journal.pone.0105544 PMC4149374 25170763

[35] De Leener B , Lévy S , Dupont SM , et al. SCT: spinal cord toolbox, an open‐source software for processing spinal cord MRI data. Neuroimage. 2017;145:24‐43. 27720818 10.1016/j.neuroimage.2016.10.009

[36] Datta E , Papinutto N , Schlaeger R , Zhu A , Carballido‐Gamio J , Henry RG . Gray matter segmentation of the spinal cord with active contours in MR images. Neuroimage. 2017;147:788‐799. 27495383 10.1016/j.neuroimage.2016.07.062

[37] Prados F , Ashburner J , Blaiotta C , et al. Spinal cord grey matter segmentation challenge. Neuroimage. 2017;152:312‐329. 28286318 10.1016/j.neuroimage.2017.03.010 PMC5440179

[38] Perone CS , Calabrese E , Cohen‐Adad J . Spinal cord gray matter segmentation using deep dilated convolutions. Sci Rep. 2018;8:1‐13. 29654236 10.1038/s41598-018-24304-3 PMC5899127

