# Supplementary material for: Cervical spinal cord atrophy in amyotrophic lateral sclerosis across disease stages
Source: Ann Clin Transl Neurol. 2023 Jan 4;10(2):213–24. doi: 10.1002/acn3.51712 (PMC9930423; doi:10.1002/acn3.51712)

**Material and Methods**

To assess if the level of the spinal cord involved at the time of MRI acquisition played a role in SC atrophy, the Kruskal-Wallis test was applied to SC metrics among healthy participants and ALS patients divided in 3 groups: cervical only (n=8, upper limb involvement), lumbar only (n=12, lower limb involvement), and multiple spinal cord level involvement (n=18; bulbar+cervical, bulbar+lumbar, cervical+lumbar, bulbar+cervical+lumbar) at MRI acquisition.

We repeated this latter analysis restricting the entire cohort to the 31 spinal - lower motor neuron dominant patients to verify if these patients had a different pattern of gray and white matter atrophy at the cervical level when only upper limbs were clinically affected. For this analysis we therefore compared lower motor neuron dominant patients with some degree of upper limbs involvement (i.e. cervical, bulbar+cervical, cervical+lumbar, bulbar+cervical+lumbar) with lower motor neuron dominant patients with lower limb impairment only.

**Results**

***Normalization effectiveness***

The goal of the normalization of spinal cord areas with V-scale and canal area was removing from the data part of the inter-subject variability due to morphological and anatomical characteristics of the study participants. The normalization effectiveness can be appreciated by comparing the standard deviation of the areas of a group before and after normalization. In ***Table 2*** we report the effect of the normalization on the whole cohort and on the healthy participants only. On the whole cohort, the average standard deviation reduction at the 4 cervical levels was 10.33% for TCA and 6.28% for GMA. Considering the healthy participants only, the average standard deviation reduction at the 4 cervical levels was 15.57% for TCA and 14.09% for GMA.

***Level of spinal cord involved at the time of MRI acquisition***

On the entire cohort and for the subset of 31 spinal - lower motor neuron dominant patients, significant between-group differences among healthy participants and patients grouped for the spinal cord levels involved at the time of MRI acquisition were detected (Kruskal-Wallis test: p≤0.021). In post-hoc tests on the entire cohort, ALS patient groups with cervical and lumbar level involvement both showed a significant reduction of GMA in terms of z-score average when compared to healthy participants (p=0.011 and p=0.032, respectively) (***Supplementary Table 1; Supplementary Figure 1***). This GM atrophy was mainly localized from C3-C4 to C4-C5 segments, and probably drove the overall TCA reduction at the C3-C4 segment. No differences were identified between cervical and lumbar patients.

In addition, lower motor neuron dominant patient with some degree of upper limb involvement and without upper limb involvement both showed a significant reduction of TCA and GMA in terms of z-score average when compared to healthy participants (***Supplementary Table 2; Supplementary Figure 2***). Significant TCA, GMA and WMA reduction was detected in all segments for patients with upper limb involvement, while TCA and GMA reduction was mainly localized from C2-C3 to C4-C5 segments in patients with lower limb impairment only. Interestingly, a significant GMA difference was identified in the C5-C6 segment of lower motor neuron dominant patients with some degree of upper limb involvement when compared to lower motor neuron dominant patients with lower limb involvement only.

**Supplementary Table 1. SC metrics between healthy participants and ALS patients according to the level of the spinal cord involved at the time of MRI acquisition.** First 2 columns: Kruskal-Wallis test assessed the between-groups differences among healthy participants and ALS patients divided in 3 groups (i.e. cervical, lumbar and multi-level involvement). Last 6 columns: Dunn's Kruskal-Wallis Multiple Comparisons tests used to assess between-group differences. Bulbar patients were not included in this analysis due to the small number of cases. Results were corrected for multiple comparisons. Significant results are highlighted in bold. Abbreviations: Healthy p. = healthy participants;

|  | **Cervical vs lumbar vs multi-level vs Healthy p.** | | **Cervical vs Healthy p.** | **Lumbar vs Healthy p.** | **Multi-level vs Healthy p.** | **Cervical vs lumbar** | **Cervical vs multi-level** | **Lumbar vs multi-level** |
| --- | --- | --- | --- | --- | --- | --- | --- | --- |
|  | *χ2* | *p-value* | *p-value* | *p-value* | *p-value* | *p-value* | *p-value* | *p-value* |
| ***TCA*** |  |  |  |  |  |  |  |  |
| z-score average | 23.167 | **< 0.001** | 0.064 | 0.061 | **< 0.001** | 0.117 | 0.117 | 0.066 |
| C2-C3 | 17.503 | **0.001** | 0.263 | 0.092 | **< 0.001** | 0.694 | 0.116 | 0.122 |
| C3-C4 | 20.632 | **< 0.001** | **0.044** | **0.035** | **< 0.001** | 0.741 | 0.323 | 0.144 |
| C4-C5 | 22.063 | **< 0.001** | 0.081 | 0.085 | **< 0.001** | 0.964 | 0.088 | 0.081 |
| C5-C6 | 22.686 | **< 0.001** | 0.126 | 0.25 | **< 0.001** | 0.48 | 0.101 | **0.01** |
| ***GMA*** |  |  |  |  |  |  |  |  |
| z-score average | 28.762 | **< 0.001** | **0.011** | **0.032** | **< 0.001** | 0.244 | 0.244 | **0.038** |
| C2-C3 | 16.053 | **0.001** | 0.158 | 0.189 | **< 0.001** | 0.874 | 0.191 | 0.146 |
| C3-C4 | 27.219 | **< 0.001** | **0.001** | **0.022** | **< 0.001** | 0.369 | 0.671 | 0.138 |
| C4-C5 | 24.091 | **< 0.001** | **0.018** | **0.027** | **< 0.001** | 0.559 | 0.46 | 0.119 |
| C5-C6 | 20.490 | **< 0.001** | 0.119 | 0.282 | **< 0.001** | 0.528 | 0.158 | **0.02** |
| ***WMA*** |  |  |  |  |  |  |  |  |
| z-score average | 20.725 | **< 0.001** | 0.205 | 0.268 | **< 0.001** | 0.081 | 0.081 | **0.02** |
| C2-C3 | 15.265 | **0.002** | 0.538 | 0.448 | **0.001** | 0.868 | **0.05** | 0.067 |
| C3-C4 | 16.366 | **0.001** | 0.201 | 0.36 | **< 0.001** | 0.539 | 0.201 | 0.056 |
| C4-C5 | 22.437 | **< 0.001** | 0.689 | 0.245 | **< 0.001** | 0.548 | **0.008** | **0.021** |
| C5-C6 | 18.302 | **< 0.001** | 0.14 | 0.416 | **< 0.001** | 0.385 | 0.227 | **0.015** |

**Supplementary Table 2. SC metrics between healthy participants and lower motor neuron dominant patients with upper and lower limb involvement.** First 2 columns: Kruskal-Wallis test assessed the between-groups differences among healthy participants and lower motor neuron dominant patients divided in patients with some degree of upper limb involvement (n = 22) and with lower limb impairment only (n = 9). Results were corrected for multiple comparisons. Last 3 columns: Dunn's Kruskal-Wallis Multiple Comparisons tests used to assess between-group differences. Significant results are highlighted in bold. Abbreviations: LMN = lower motor neuron dominant; Healthy p. = healthy participants.

|  | **LMN upper limb involvement vs  LMN lower limb involvement only vs Healthy p.** | | **LMN upper limb involvement vs Healthy p.** | **LMN lower limb involvement only vs Healthy p.** | **LMN upper limb involvement vs LMN lower limb involvement only** |
| --- | --- | --- | --- | --- | --- |
|  | *χ2* | *p-value* | *p-value* | *p-value* | *p-value* |
| ***TCA*** |  |  |  |  |  |
| z-score average | 18.724 | **< 0.001** | **< 0.001** | **0.041** | 0.22 |
| C2-C3 | 11.689 | **0.003** | **0.001** | **0.04** | 0.688 |
| C3-C4 | 18.972 | **< 0.001** | **0.001** | **0.011** | 0.49 |
| C4-C5 | 15.930 | **< 0.001** | **0.001** | **0.045** | 0.328 |
| C5-C6 | 16.607 | **< 0.001** | **0.001** | 0.252 | 0.056 |
| ***GMA*** |  |  |  |  |  |
| z-score average | 24.777 | **< 0.001** | **< 0.001** | **0.029** | 0.146 |
| C2-C3 | 12.086 | **0.002** | **0.003** | **0.023** | 0.955 |
| C3-C4 | 24.123 | **< 0.001** | **0.001** | **0.011** | 0.308 |
| C4-C5 | 22.158 | **< 0.001** | **0.001** | **0.033** | 0.186 |
| C5-C6 | 17.547 | **< 0.001** | **0.001** | 0.608 | **0.015** |
| ***WMA*** |  |  |  |  |  |
| z-score average | 13.895 | **0.001** | **0.001** | 0.161 | 0.218 |
| C2-C3 | 7.690 | **0.021** | **0.017** | 0.314 | 0.387 |
| C3-C4 | 11.421 | **0.003** | **0.002** | 0.124 | 0.408 |
| C4-C5 | 10.436 | **0.005** | **0.004** | 0.168 | 0.375 |
| C5-C6 | 12.528 | **0.002** | **0.001** | 0.345 | 0.121 |

**Supplementary Figure 1. SC metrics for patients grouped according to the level of the spinal cord involved at the time of MRI acquisition.** Boxplot of TCA, GMA and WMA as z-score average for healthy participants and ALS patients grouped according to the level of the spinal cord involved at the time of MRI acquisition.


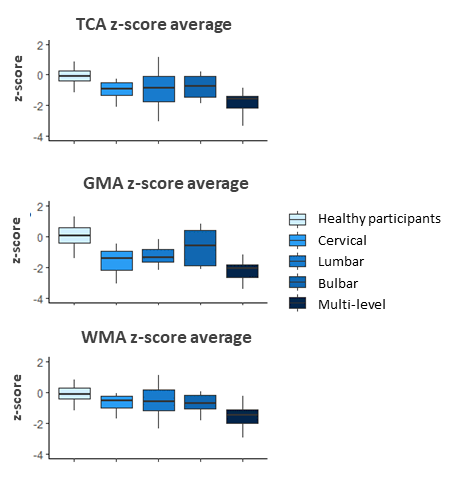


**Supplementary Figure 2. SC metrics for lower motor neuron dominant patients with upper and lower limb involvement at the time of MRI acquisition.** Boxplot of TCA, GMA and WMA as z-score average for healthy participants and lower motor neuron dominant patients divided in patients with some degree of upper limb involvement and with lower limb impairment only.


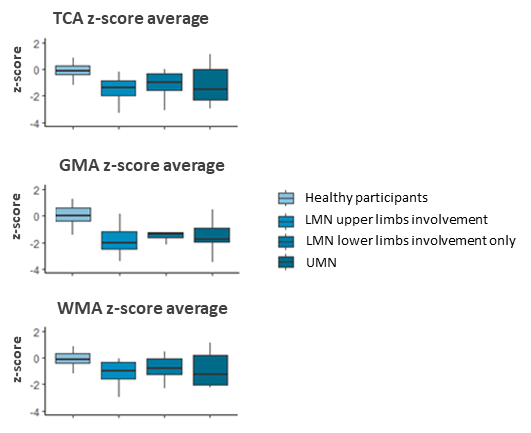

Supplement: Supplementary file 1 — Table S1. SC metrics between healthy participants and ALS patients according to the level of the spinal cord involved at the time of MRI acquisition. Table S2. SC metrics between healthy participants and lower motor neuron dominant patients with upper and lower limb involvement. Figure S1. SC metrics for patients grouped according to the level of the spinal cord involved at the time of MRI acquisition. Figure S2. SC metrics for lower motor neuron dominant patients with upper and lower limb involvement at the time of MRI acquisition. [file ACN3-10-213-s001.docx]
